# The expression of cytokines in the aqueous humor and serum during endotoxin-induced uveitis in C3H/HeN mice

**Published:** 2010-08-21

**Authors:** Yingzhi Xu, Wei Chen, Hong Lu, Xiaofeng Hu, Shang Li, Jing Wang, Li Zhao

**Affiliations:** 1 Department of Ophthalmology, Beijing Chaoyang Hospital, Capital Medical University, Beijing, China; 2 Eye Department, Beijing Haidian Maternal and Child Health Hospital, Beijing, China

## Abstract

**Purpose:**

The cytokines present in the aqueous humor and serum of C3H/HeN mice with endotoxin-induced acute anterior uveitis were analyzed, and the potential role of the cytokines in the pathogenesis of the disease was investigated.

**Methods:**

One hundred and eighty C3H/HeN mice were divided into an experimental group (n=150) and a control group (n=30). The mice in the experimental group were footpad-injected with 200 μg *Vibro cholerae* endotoxin (classical biotype, serotype Ogawa). The mice were then executed 4 h, 8 h, 16 h, 24 h, and 48 h after the injection of endotoxin. Aqueous humor and peripheral blood samples were collected using a microinjector. Ten samples were pooled together for analysis and centrifuged at 705× g, at 4 °C, for 3 min. The supernatant was collected and stored at −80 °C. The concentrations of tumor necrosis factor-α (TNF-α), interleukin-1 (IL-1), interleukin-6 (IL-6), interleukin-10 (IL-10), and interferon-γ (IFN-γ) in the samples were measured using a Cytometric Bead Array (CBA).

**Results:**

Acute anterior uveitis was successfully induced in C3H/HeN mice. At roughly 16 h post-injection, the concentrations of both IL-1 and IL-6 reached peak levels, and were significantly different from the control group (p=0.001 and p=0.001, respectively). At 24 h post-injection, the concentrations of IFN-γ and IL-10 in the aqueous humor reached peak levels, and were significantly different from the control group (p=0.022 and p=0.003). The concentrations of IFN-γ in serum at 4 and 24 hours were significantly different from the control group (p=0.033 and p=0.032). The concentration of IL-10 in serum, at 24 h post-injection, was also found to be significantly different from the control group (p=0.003). The cytokine expression levels in the aqueous humor were consistent with what would be expected during the process of inflammation.

**Conclusions:**

Both IL-1 and IL-6 appear to play an important role in acute anterior uveitis; furthermore, the severity of inflammation may be associated with the dynamic balance of IFN-γ and IL-10. Our results suggest that the cytokine network might be a useful therapeutic target in the treatment of acute anterior uveitis.

## Introduction

Acute anterior uveitis (AAU) is the most common form of uveitis. In the past few years, inflammation has been recognized as a major driving force of AAU. It is now well established that, starting from the initial lesion to the iris and the aqueous humor in the eye, numerous cellular and molecular inflammatory components participate in the disease process. Monocyte-derived macrophages and T-lymphocytes are the predominant invading immune cells found in evolving lesions. Both cell types produce a wide array of soluble inflammatory mediators (cytokines and chemokines) that are critically important in the initiation and perpetuation of the disease [1].

Recent studies suggest that the pathogenesis of AAU is related to the expression of toll-like receptor-4 (TLR-4). Activation of TLR4 on macrophages by lipopolysaccharide (LPS) promotes activation of the transcription factor nuclear nuclear factor-κB (NF-κB) through an immunostimulatory intracellular signaling pathway. Consequently, the resulting induction of various proinflammatory cytokines, chemokines, and antimicrobial activities initiates a rapid inflammatory response designed to eliminate the invading pathogens, and is characterized by the recruitment of leukocytes to the site of infection [2]. In this study, we established an endotoxin-induced uveitis (EIU) model in C3H/HeN mice through injection of *Vibrio cholerae* endotoxin (lipopolysaccharide (LPS)). EIU is a well-established experimental model for AAU that is used with several different species, including Wistar rats [3]. Using cytometric bead array technology, concentrations of the cytokines tumor necrosis factor-α (TNF-α), interleukin-1 (IL-1), interleukin-6 (IL-6), interleukin-10 (IL-10), and interferon-γ ­(IFN-γ) in aqueous humor and serum were analyzed in cases of endotoxin-induced acute anterior uveitis in C3H/HeN mice. The resulting data imply that cytokines are potentially associated with the pathogenesis of AAU and could be used as indicators for improved early detection, prevention, and treatment of the disease.

## Methods

### Apparatus used in cytometric bead array

BD^™^ Flow cytometry (FACSCalibur, BD Company, San Diego, CA).

BD^™^ Cell Quest and CBA software (material number 550065; BD Company).

BD^™^ CBA Mouse TNF, IL-1, IL-6, IL-10, and IFN-γ Flex Set (material number 558266; BD Company).

### Animals

One hundred and eighty C3H/HeN male mice, 6 to 8 weeks old and weighing between 16 g and 25 g, were obtained from the Beijing Wei Tong Li Hua Laboratory (Animal Victoria Limited, Beijing, China). The mice were divided into an experimental group (n=150) and a control group (n=30). The experimental group was further divided into five subgroups of 30 mice each, according to post-LPS injection time points (4 h, 8 h, 16 h, 24 h, and 48 h).

### Establishing the endotoxin-induced uveitis (EIU) model

The experimental group was footpad-injected with 200 μg *Vibro cholerae* endotoxin [4] (classical biotype, serotype Ogawa) dissolved in 0.1 ml normal saline. The mice were examined by slit lamp in groups of 30 the time points of 4 h, 8 h, 16 h, 24 h, and 48 h after injection of endotoxin. The control group was footpad-injected with 100 μl saline solution. Clinical manifestations were evaluated according to the protocol described by Lajavaidi [5]. The intensity of clinical ocular inflammation was scored on a scale from 0 to 4 for  each eye, derived from a scale described previously as follows: 0, no sign of inflammation; 1, discrete inflammation in iris and conjunctiva; 2, dilatation of iris and conjunctiva vessels; 3, hyperemia in iris associated with Tyndall effect in anterior chamber; 4, same signs as in 3, but a point was added if synechia or fibrin was observed.

### Cytokine detection

#### Sample collection

The mice were executed in groups of 30 at 4 h, 8 h, 16 h, 24 h, and 48 h after injection of endotoxin. The aqueous humor and peripheral blood samples were collected using a microinjector. Ten samples were pooled for analysis and centrifuged at 705× G, at 4 °C, for 3 min. The supernatant was collected and stored at −80 °C. The concentrations of TNF-α, IL-1, IL-6, IL-10, and IFN-γ in the aqueous humor and serum were quantified using a cytometric bead array (CBA).

#### Generation of standard curves

The cytokine standard solutions included with the BD CBA Flex Sets were serially diluted to the following concentrations: 2,500 μg/l, 1,250 μg/l, 625 μg/l, 312 μg/l, 156 μg/l, 80 μg/l, 40 μg/l, 20 μg/l, and 0 μg/l. A control sample was also included in the measurements. Measurements for the different concentrations were obtained using the FACSCalibur, and standard curves were generated using the BD CBA software.

### Sample preparation and analysis

A total of 50 μl of serum was mixed with 50 μl of capture beads and 50 μl of PE-labeled detection antibodies. After incubation in the dark at room temperature for 2 h, 1 ml of soluble protein master buffer lotion was added. The sample was then centrifuged at 200× g for 5 min and washed twice, before the addition of 300 μl buffer lotion. Results were obtained using BD CellQuest and BD CBA software.

### Statistics

Data was analyzed using paired *t* tests, performed using SPSS (version 13.0; SPSS Inc., Chicago, IL) statistical software. All data were expressed as the means, plus or minus standard deviation. A p value of 0.05 or less was interpreted as indicating statistical significance when comparing experimental and control groups.

## Results

**Figure 1:**
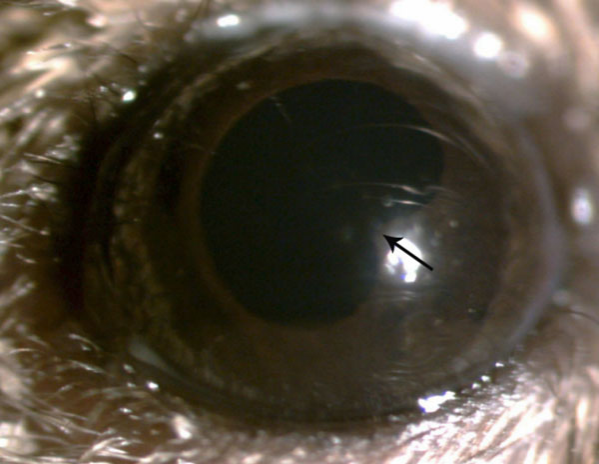
Clinical manifestations of endotoxin-induced uveitis in C3H/HeN mice. The image shows the eye of an animal 24 h after injection with lipopolysaccharides. The arrow points out the pupil adhesion.

**Figure 2:**
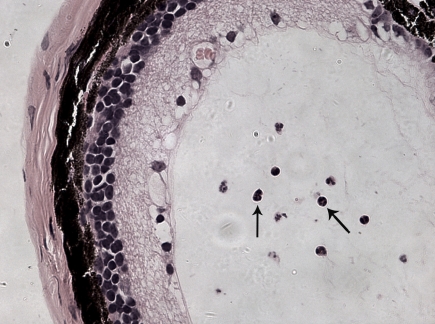
Hematoxylin-eosin stain study for neutrophils in the aqueous humor. The image shows the eye 24 h after injection of lipopolysaccharides. Arrows indicate neutrophils in the anterior chamber.

AAU was induced in 100% of the C3H/HeN mice that were footpad-injected with 200 μg LPS (Figure 1 and Figure 2). The inflammatory reaction reached its peak between 16 h and 24 h, and subsided after 48 h (Table 1).

**Table 1:** Clinical scores of LPS-induced mice at various time points.

**Time**	**C3H/HeN(LPS)**	**control**
0 h	0±0	0±0
4 h	1.0±0.0	0±0
8 h	1.3±0.3	0±0
16 h	2.0±0.7	0±0
24 h	3.8±0.4	0±0
48 h	3.3±0.5	0±0

**Figure 3:**
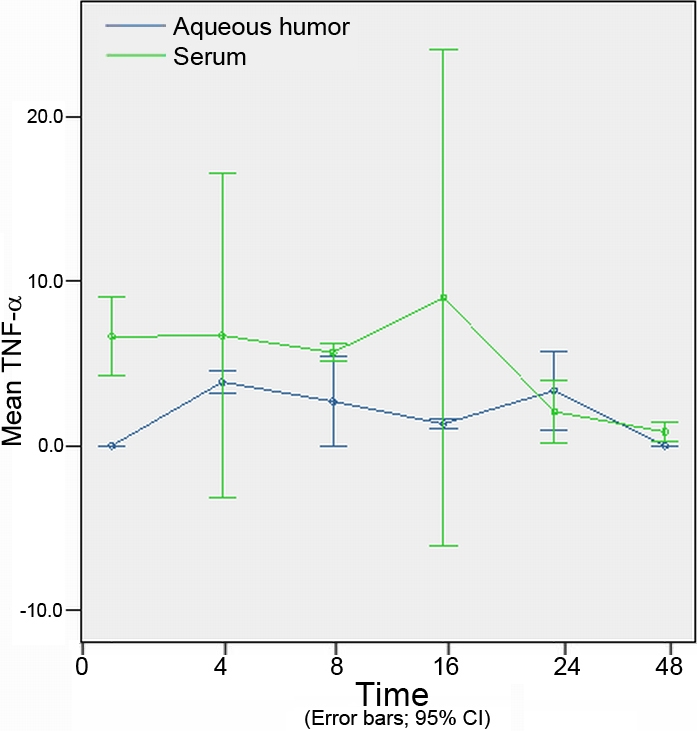
The expression levels tumor necrosis factor-α in the aqueous humor and serum at different time points (expressed as the mean μg/l±SD). The concentration of tumor necrosis factor-α in the aqueous humor reached a peak at 4 h. In the serum, tumor necrosis factor-α reached a peak concentration 16 h after injection. After 48 h, tumor necrosis factor-α concentrations in both the aqueous humor and the serum returned to their original levels.

**Figure 4:**
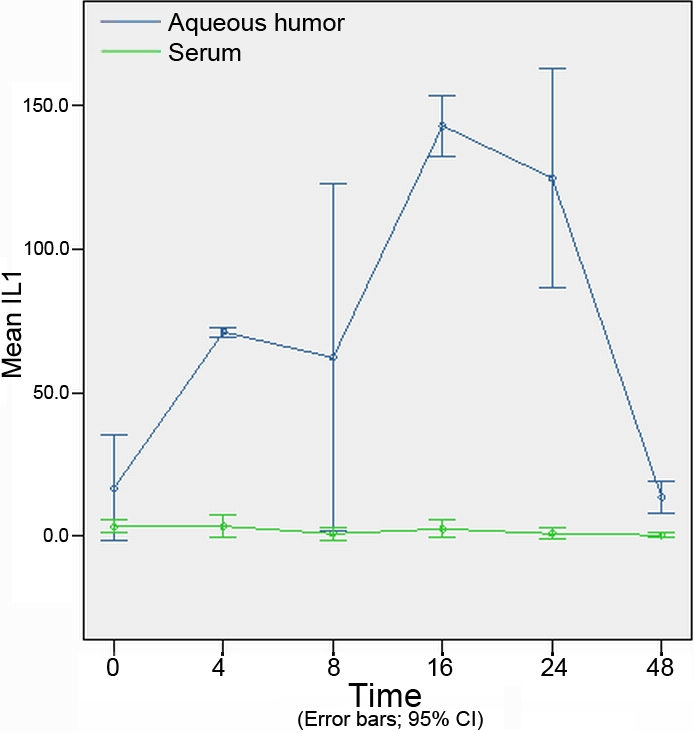
The expression levels of interleukin-1 in the aqueous humor and serum at different time points (expressed as the mean μg/l±SD). The concentration of interleukin-1 in the aqueous humor increased significantly at 4 h, reached a peak at 16 h, and finally returning to its original concentration at 48 h. But it showed little difference in the serum.

**Figure 5:**
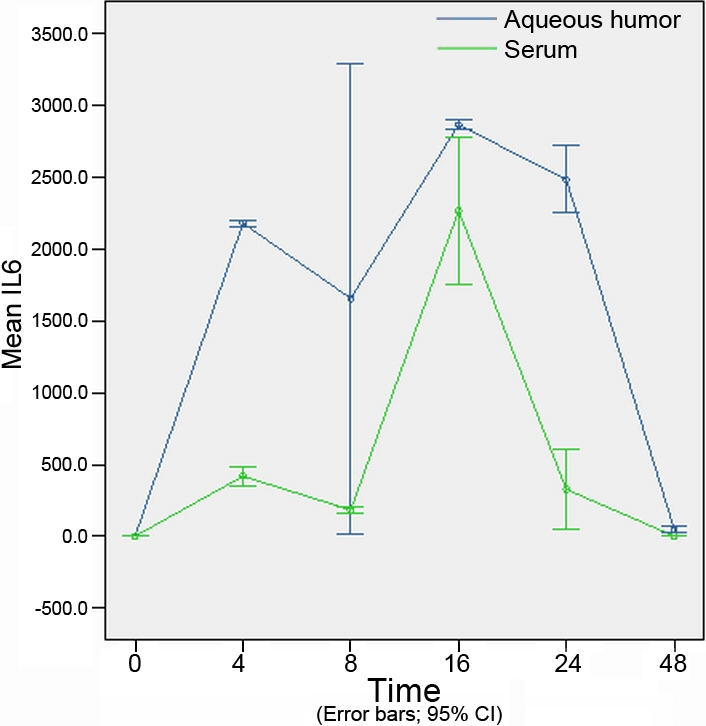
The expression of interleukin-6 in the aqueous humor and serum at different time points (expressed as the mean μg/l±SD). The concentration of interleukin-6 in the aqueous humor was consistently much higher than in the serum. The interleukin-6 concentration in the aqueous humor rapidly increased in the first 4 h, reached a peak at 16 h, and returned to the original concentration after 48 h.

**Figure 6:**
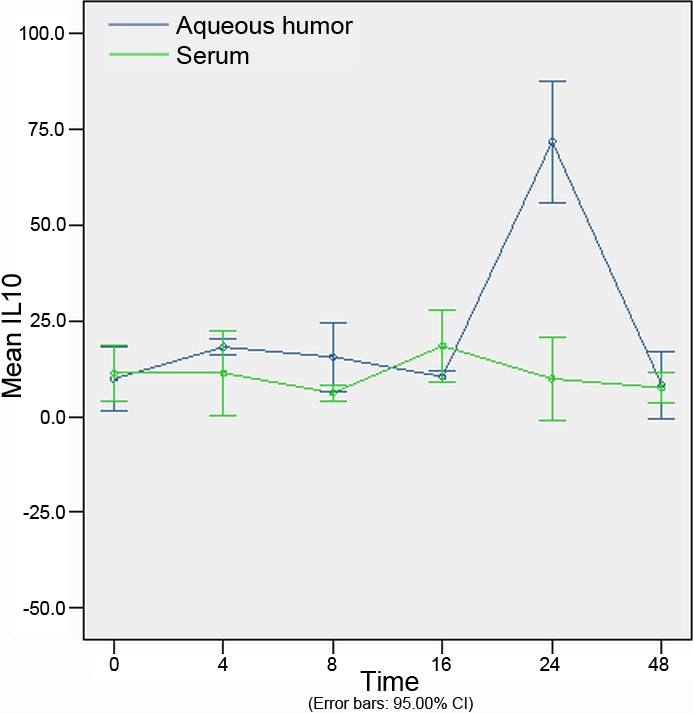
The expression of interleukin-10 in the aqueous humor and serum at different time points (expressed as the mean μg/l±SD). Interleukin-10 reached a peak concentration at 24 h, and returned to its original concentration at 48 h in aqueous humor. The concentration of interleukin-10 observed in the serum was similar to that in the aqueous humor, increasing at 8 h and reaching a maximum concentration at 16 h.

**Figure 7:**
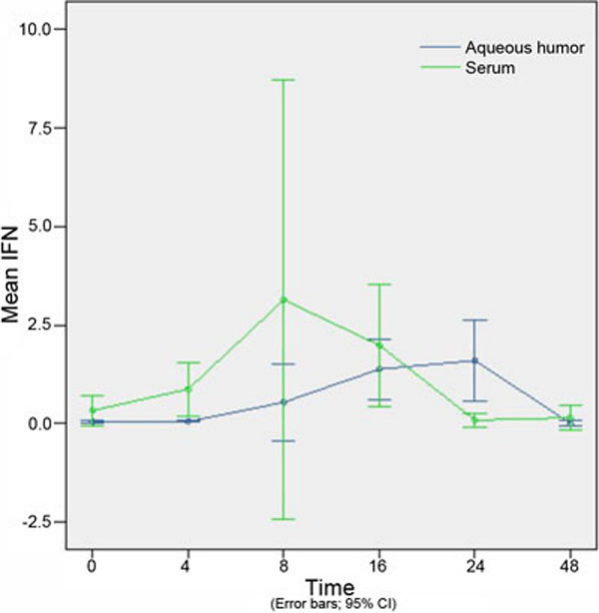
The expression of interferon-γ in the aqueous humor and serum at different time points (expressed as the mean μg/l±SD). Interferon-γ concentrations in the aqueous humor and serum were not significantly different at 8 h or 16 h in comparison to the control group.

The concentration of cytokines at different time points in the aqueous humor are shown in Table 2. The concentrations of cytokines at different time points in the serum are shown in Table 3. Concentration of cytokines of different experimental groups in the aqueous humor and serum are shown in Figure 3, Figure 4, Figure 5, Figure 6, and Figure 7.

**Table 2:** Cytokine levels in the aqueous humor of each group at various time points (expressed as the mean μg/l±SD).

**Group**	**TNF-α**	**IL-1**	**IL-6**	**IL-10**	**IFN-γ**
control	0.000±0.0000	16.800±7.4030	0.300±0.0985	9.910±3.3717	0.053±0.0116
4 h	3.870±0.2667	71.197±0.6623	2180.663±8.0023	18.203±0.8829	0.060±0.0100
8 h	2.693±1.105	62.243±24.4131	1656.800±658.6320	15.583±0.3.5814	0.547±0.3946
16 h	1.337±0.1102	142.990±4.2623	2867.140±14.1790	10.440±0.6056	1.387±0.3099
24 h	3.360±0.9694	124.790±15.2831	2485.210±94.1627	71.843±6.3632	1.603±0.04153
48 h	0.000±0.0000	13.630±2.2104	51.410±8.6309	8.367±3.5473	0.013±0.0231

**Table 3:** Cytokine levels in the serum of EIU mice at various time points (expressed as the mean μg/l±SD).

**Group**	**TNF-α**	**IL-1**	**IL-6**	**IL-10**	**IFN-γ**
control	6.623±0.9552	3.437±0.8765	1.270±0.6102	11.377±2.9502	0.337±0.1554
4 h	6.697±3.94 9	3.487±1.5084	418.747±27.1557	11.397±4.4626	0.880±0.2740
8 h	5.667±0.2023	0.877±0.8639	183.477±9.7054	6.183±0.8262	3.143±2.2402
16 h	9.000±6.0536	2.563±1.2303	2269.423±206.1097	18.477±3.7873	1.993±0.6229
24 h	2.070±0.7590	0.937±0.7742	327.170±112.0372	9.903±4.3021	0.090±0.0700
48 h	0.853±0.2479	0.410±0.3995	1.333±0.5463	7.570±1.6452	0.153±0.1305

### Concentration of tumor necrosis factor-α in aqueous humor and serum

The initial concentration of TNF-α was lower in the aqueous humor than in the serum. After injection of LPS, the concentration of TNF-α in the aqueous humor rapidly increased, reaching a peak 4 h after injection, which was found to be statistically significant in comparison with the control group (p=0.002). In the serum, TNF-α reached a peak concentration 16 h after injection, though it was not found to be statistically significant in comparison to the control group (p=0.555). After 48 h, TNF-α concentrations in both the aqueous humor and the serum returned to their original levels. No significant differences between the TNF-α concentrations in the aqueous humor and the serum were found at any time points (Figure 3).

### Concentration of interleukin-1 in aqueous humor and serum

Upon injection with endotoxin, IL-1 expression levels changed dramatically in the aqueous humor, but showed little difference in the serum. Compared with the control group, the concentration of IL-1 in the aqueous humor had increased significantly at 4 h (p=0.006), reached a peak (p=0.001) at 16 h, remained at an increased concentration (p=0.012) through the 24 h time point. It had finally returned to its original concentration at 48 h (Figure 4).

### Concentration of interleukin-6 in aqueous humor and serum

Similar changes in the concentration levels of IL-6 were observed in the aqueous humor and the serum. Significant differences were observed at 4 h, 16 h, and 24 h (p=0.000, p=0.042, and p=0.003, respectively) when compared with the control group. Notably, the concentration of IL-6 in the aqueous humor was consistently much higher than in the serum. The IL-6 concentration in the aqueous humor rapidly increased and was much higher than in the serum during the first 4 h (p=0.000), reaching a peak at 16 h (p=0.000), and returning to the original concentration after 48 h (p=0.009; Figure 5).

### Concentration of interleukin-10 in aqueous humor and serum

No significant differences in the concentration of IL-10 in the aqueous humor were found at 4 h, 8 h, or 16 h (p=0.055, p=0.057, and p=0.817, respectively) between the endotoxin-injected and control groups. IL-10 reached a peak concentration at 24 h that was found to be statistically significant in comparison with the control group (p=0.003), and returned to its original concentration at 48 h (p=0.602). The concentration of IL-10 observed in the serum was similar to that in the aqueous humor, increasing at 8 h and reaching a maximum concentration at 16 h. When compared with the control group, no significant difference was found at any time point (p>0.05; Figure 6).

### Concentration of interferon-γ in aqueous humor and serum

IFN-γ concentrations in the aqueous humor and serum were not significantly different at 8 h or 16 h, in comparison with the control group (p=0.138 and p=0.377, respectively); however, statistically significant differences were observed at the 4 h and 24 h time points (p=0.033 and p=0.032, respectively; Figure 7).

## Discussion

Although the disease mechanisms of AAU have been extensively studied in recent years, in particular by the Luhong’s research group [6], its pathogenesis remains poorly understood. Recent research, however, suggests that TLR4 signaling pathways might be involved in the pathogenesis of the disease. To further elucidate the role of TLR4 in AAU, we chose to study C3H/HeN and C3H/HeJ (*TLR4* gene-deficient) mice in this work. EIU is a well-established experimental model for AAU. Here, we characterized EIU according to the secreted proteins in the anterior chamber 4 h after injection with LPS, and the exudative membrane in the pupil area 12-16 h after injection with LPS, respectively. The inflammatory reaction reached a peak between 18 h and 24 h after injection, and subsided after 48 h. AAU was successfully induced in C3H/HeN mice by a footpad injection of 200 μg *Vibrio cholerae* LPS.

Recent studies have shown that cytokines play an important role in the pathogenesis of AAU [3]. Many reports have demonstrated that treatment with cytokines such as IL-10, IL-4, and the neutralizing antibody of TNF can aid in the treatment of the disease. However, the biologic activity of cytokines is complex; thus, it is necessary to evaluate the efficacy of cytokines in vivo.

To investigate the role of cytokines in the pathogenesis of AAU, we used a *Vibrio cholerae* LPS-induced-C3H/HeN mouse EIU model and measured the concentrations of TNF-α, IL-1, IL-6, IL-10, and IFN-γ in both the aqueous humor and serum at various time points after injection with LPS.

Our study provides novel evidence of changes in aqueous humor and serum cytokine concentrations in C3H/HeN mice over the course of AAU. TNF-α reached a peak concentration in the early stages of inflammation; thus, TNF-α might play an important role in the initiation of the inflammatory response. It was also found that the levels of TNF in both the serum and the aqueous humor were significantly higher in the early stages of EIU in comparison with the control group. Other reports have indicated that TNF-α concentrations in the aqueous humor increase before the clinical signs of uveitis can be observed. Koizumi et al. [7] found evidence of leukocyte migration, retinal vein endothelial cell apoptosis, and vascular leakage in EIU rats. It has been reported that treatment with TNFR-IgG can significantly reduce these responses.

Perez-Guijo [8] and Santos-Lacomba [9] reported that the concentrations of TNF-α in the serum and aqueous humor of uveitis patients were significantly higher than those of a normal control group. The recurrence of uveitis is often associated with elevated serum levels of TNF-α with significant changes in aqueous humor levels. Likewise, our study found that the expression levels of TNF-α and IFN-γ in the serum were significantly higher than those in the aqueous humor. Taken together, this evidence indicates that TNF-α plays an important role in the etiology of uveitis.

IL-1, IL-6, and IL-10 reached maximum concentrations during the peak stage of inflammation, and concentrations were consistently lower in the serum than in the aqueous humor. This suggests that IL-1, IL-6, and IL-10 are produced in an autocrine fashion by the iris, rather than being products of damage to the blood–aqueous barrier. It has been previously reported that expression of *IL-6* mRNA was detected; this indicates that IL-6 was produced in an autocrine fashion by local tissues. IL-1 expression levels in the aqueous humor were significantly higher than those in the serum, indicating that autocrine production of IL-1 was associated with acute anterior uveitis. IL-6 levels reached a minor peak 4 h after injection with LPS; this could be related to the rapid increase of TNF-a, IFN-γ, and IL-1 that was observed. Possibly due to its inhibitory effect on other cytokines, the IL-10 response was different from the other cytokines analyzed. IL-10 can regulate both the synthesis of inhibitory cytokines and antigen presentation through the coexpression of transcription factors Spl and Sp2 [10], thereby playing an important anti-inflammatory role. IL-10 has also been shown to be a positive regulator of IL-l receptor antagonists [11]. Muzio et al. [12] found that IL-10 downregulated expression of toll-like receptor-4 (TLR4), a receptor that mediates the innate immune response through the LPS signal transduction pathway. IL-10, together with IL-2, also promotes the differentiation of cytotoxic T-Lymphocytes (cTL) [13-15]. Matta Bharati et al. [16] reported that the expression levels of IL-10 and TGF-β2 increased, while levels of TNF-α, IFN-γ, and IL-2 decreased in a tolerance-induced EIU model. Hisashi Mashimo [4] found that continuously high expression levels of IL-10 in the eye and a reduction of peripheral blood neutrophil chemotaxis both played significant roles in the mechanism of LPS tolerance in a rat model of footpad-injection EIU.

AAU is closely related to inflammatory cytokines produced by Thl-type cellular immune responses, such as IL-l, TNF-a, and IFN-γ. An excessive Thl response could lead to contact hypersensitivity and cell-mediated autoimmune responses [17]. Th2 cells can produce inhibitory cytokines such as IL-10, and reduce pathological damage to eye tissue. Further investigation into the interaction and dynamic changes of cytokines may provide insights into the pathogenesis of acute anterior uveitis, potentially leading to more-effective treatments.
